# Reuse of disposable syringes and needles in patients with type 2 diabetes

**DOI:** 10.1186/1758-5996-7-S1-A189

**Published:** 2015-11-11

**Authors:** Cátia Moreira Guterres, Guilherme Alcides Flores Soares Rollin, Rodrigo Antonini Ribeiro, Gisele Nader Bastos, Karine Margarites Lima, Fabiano Barrionuevo, Luciano Serpa Hammes, Maria Claudia Schardosim Cotta de Souza, Tássia Scholante Delabary, Leni Everson Araújo Leite, Rubia Natasha Maestri, Carolina Robinson, Maicon Falavigna, Regina Kuhmmer

**Affiliations:** 1 Hospital Moinhos de Vento, Porto Alegre, Brazil

## Background

Despite the recommendation of manufacturers for single-use of syringes and needles for insulin administration, most patients reuse these devices, a practice supported by national guidelines.

## Objectives

To estimate the frequency of needles and syringes reuse for insulin administration by patients with type 2 DM. In addition, to review patients' practices related to insulin administration.

## Materials and methods

Cross-sectional study in an emergency department of a public hospital, in Porto Alegre, Brazil. We assessed sociodemographic and clinical variables related to the management of diabetes; physical examination was performed to evaluate the presence of lipodystrophy, injection site infection and hematomas.

## Results

From October 2014 to January 2015, we included 28 participants. Fifteen (54%) were female, average age was 67 (SD 14) yrs. and average BMI was 30 kg/m2 (SD 8). Household monthly income was less than R$ 1.000 (US$ 317) for 68% of the participants. Median time of insulin therapy was 10 yrs. (range 6 to 20 yrs.); 23 (74%) self-administered insulin injections. Twenty-seven (96%) participants receive needles, syringes and insulin from public health system. Reuse of disposable syringes and needles was reported by 75% of participants. The frequency of re-utilization of needles and syringes ranged from two to 21 times; the median re-use frequency was three (IQR 3 to 7.5). Main reasons for syringe and needle changes was pain (54%), guidance of a health professional (14%) and blunt needle (14%). Twenty-two subjects (79%) reported not having received any guidance from a health professional regarding the reuse of needles and syringes. Only 16 (57%) of the participants disinfect with alcohol the injection site; hand-washing before insulin-administration was reported by 26 (93%) subjects. Although not recommended, twelve (43%) disinfect the needle with alcohol for reuse. Needlesticks injures due to syringes and needles reuse were reported by 13 (46%) participants. Lipohypertrophy was present in two (7%), hematomas in five (18%), and one subject had injection site infection at the time of the evaluation.

## Conclusion

Reuse rates are high and complications, such as lipohypertrophy and hematomas, are frequent. However, most participants had not received adequate guidance on syringes and needles re-utilization. It is important that healthcare professionals provide adequate guidance for patients with type 2 DM that are likely to reuse syringes and needles.

